# The Acceptability, Feasibility, and Utility of Portable Electroencephalography to Study Resting-State Neurophysiology in Rural Communities

**DOI:** 10.3389/fnhum.2022.802764

**Published:** 2022-03-21

**Authors:** Supriya Bhavnani, Dhanya Parameshwaran, Kamal Kant Sharma, Debarati Mukherjee, Gauri Divan, Vikram Patel, Tara C. Thiagarajan

**Affiliations:** ^1^Child Development Group, Sangath, Goa, India; ^2^Public Health Foundation of India, New Delhi, India; ^3^Sapien Labs, Chennai, India; ^4^Indian Institute of Public Health-Bengaluru, Public Health Foundation of India, Bengaluru, India; ^5^Department of Global Health and Social Medicine, Harvard Medical School, Boston, MA, United States; ^6^Department of Global Health and Population, Harvard T.H. Chan School of Public Health, Boston, MA, United States; ^7^Sapien Labs, Arlington, VA, United States

**Keywords:** resting-state EEG, preschool children, LMIC, EMOTIV, portable EEG

## Abstract

Electroencephalography (EEG) provides a non-invasive means to advancing our understanding of the development and function of the brain. However, the majority of the world’s population residing in low and middle income countries has historically been limited from contributing to, and thereby benefiting from, such neurophysiological research, due to lack of scalable validated methods of EEG data collection. In this study, we establish a standard operating protocol to collect approximately 3 min each of eyes-open and eyes-closed resting-state EEG data using a low-cost portable EEG device in rural households through formative work in the community. We then evaluate the acceptability of these EEG assessments to young children and feasibility of administering them through non-specialist workers. Finally, we describe properties of the EEG recordings obtained using this novel approach to EEG data collection. The formative phase was conducted with 9 families which informed protocols for consenting, child engagement strategies and data collection. The protocol was then implemented on 1265 families. 977 children (Mean age = 38.8 months, SD = 0.9) and 1199 adults (Mean age = 27.0 years, SD = 4) provided resting-state data for this study. 259 children refused to wear the EEG cap or removed it, and 58 children refused the eyes-closed recording session. Hardware or software issues were experienced during 30 and 25 recordings in eyes-open and eyes-closed conditions respectively. Disturbances during the recording sessions were rare and included participants moving their heads, touching the EEG headset with their hands, opening their eyes within the eyes-closed recording session, and presence of loud sounds in the testing environment. Similar to findings in laboratory-based studies from high-income settings, the percentage of recordings which showed an alpha peak was higher in eyes-closed than eyes-open condition, with the peak occurring most frequently in electrodes at O1 and O2 positions, and the mean frequency of the alpha peak was found to be lower in children (8.43 Hz, SD = 1.73) as compared to adults (10.71 Hz, SD = 3.96). We observed a deterioration in the EEG signal with prolonged device usage. This study demonstrates the acceptability, feasibility and utility of conducting EEG research at scale in a rural low-resource community, while highlighting its potential limitations, and offers the impetus needed to further refine the methods and devices and validate such scalable methods to overcome existing research inequity.

## Introduction

Electroencephalography (EEG) is a non-invasive technology designed to capture electrical signals from the brain at high temporal resolution. It has contributed to our understanding of brain development and function across the lifespan for decades. EEG can be recorded while participants are in a resting-state condition, either with their eyes closed or open, or while they are performing specific tasks. A variety of metrics derived from EEG traces obtained during these paradigms have been tested for their ability to index cognitive and social development and function in children and adults (Deary et al., 2010; Loo et al., 2016; Anderson and Perone, 2018; Bhavnani et al., 2021). Evidence is also beginning to emerge of the potential of EEG metrics to enable early identification of children at risk for developmental disorders (Bosl et al., 2011, 2018; Snyder et al., 2015; Gurau et al., 2017).

Historically though, most EEG research has been conducted in highly controlled settings of laboratories based in high-income settings, with little ethnic diversity amongst study participants (Bhavnani et al., 2021). Given emerging evidence that some EEG metrics might be sensitive to cultural and contextual differences across global settings (Knyazev et al., 2012; Alahmadi et al., 2016; Parameshwaran et al., 2021), it is critical to diversify the population of participants from whom inferences on brain development and function are drawn and ensure that low and middle income countries (LAMIC), in which the majority of the world’s population resides, are well represented in this important field of research (Valdes-Sosa et al., 2021). Furthermore, these populations are the most likely to benefit from such research, since they reside in countries with a high prevalence of risk factors that adversely impact the developing brain and have the highest number of children with neurodevelopmental disorders (Lu et al., 2016; Bitta et al., 2017), making it imperative to test the generalizability of the evidence that has accumulated from research in high-income settings.

Developmental EEG research studies have begun to emerge from LAMIC such as Bangladesh, The Gambia and Malawi (Leal Storrs, 2017; Jensen et al., 2019; Katus et al., 2019; Xie et al., 2019; Neto et al., 2021). This has been possible, in part, because research personnel in such settings have been trained to collect EEG data. However, in order to achieve the vision of using EEG at scale in global settings, neurophysiological research has to reduce its dependence on prohibitively expensive equipment and highly trained personnel such as technicians, Ph.D. students and post-doctoral researchers. EEG data collection methods also need to be amenable to being implemented in uncontrolled settings such as community centers or even households. These advances are crucial to harnessing the potential of this technology in contributing to our understanding of the typical and atypical brain toward improving the health of populations.

Encouragingly, low-cost and portable EEG devices are already available in the market through companies such as EMOTIV^1^, Cognionics^2^, and OpenBCI^3^. Most companies offer user-friendly interfaces for ease of data collection, as well as services related to cloud storage and computing of collected data, thereby reducing the need for technical expertise at the site of data collection. The time is ripe to test the potential of the portable version of EEG technology to be taken to scale in low resource settings. Evidence is beginning to accumulate on the feasibility and validity of using portable EEG devices in research studies (Ekandem et al., 2012; Badcock et al., 2013; De Vos and Debener, 2014; Lau-Zhu et al., 2019). Most of this data has emerged from studies conducted on adult participants, including studies from our group which have demonstrated the utility of the EPOC device to collect data from adults in remote rural settings in India (Parameshwaran et al., 2021). However, to our knowledge, there are no published reports demonstrating the utility of the EMOTIV EPOC device to collect EEG data on a large population-based sample of preschool children in low resource settings.

Therefore, the aims of this study were (a) to establish a standard operating protocol to collect resting-state EEG data using a low-cost portable EEG device in rural households through formative work in the community, (b) to evaluate the acceptability of conducting resting-state EEG assessments on 3-year-old children, (c) to evaluate the feasibility of administering these EEG assessments through non-specialist workers in a large sample of children and adults, and (d) to describe properties of the EEG recordings obtained using this novel approach to EEG data collection.

## Materials and Methods

### Study Site and Ethics

This work was nested within a larger follow-up study of the participants of the SPRING (Sustainable Program Incorporating Nutrition and Games) cluster randomized controlled trial, conducted in 120 villages in Rewari district in rural Haryana, India, when children turned 3 years of age. Rewari district is predominantly agricultural (70% rural households) with some industrial sectors (“National Family Health Survey” n.d.). While almost all households have electricity (98.6%) and improved drinking water sources such as piped water or public taps (93.5%), fewer have non-shared toilet facilities (69.7%) and use clean fuel for cooking (39.1%) (“National Family Health Survey” n.d.). The SPRING trial tested an intervention designed to optimize growth and development in early childhood, and has been described in detail elsewhere (registered with Clinical-Trials.gov, number NCT02059863) (Lingam et al., 2014; Divan et al., 2015; Bhopal et al., 2019a,b).

This study was conducted in accordance with the Declaration of Helsinki. Parents provided written informed consent before their own and their child’s participation in this study. The study received approval from institutional review boards of the Public Health Foundation of India (18 July 2018) and Sangath (23 August 2018).

### Field Personnel

**FIGURE 1:**
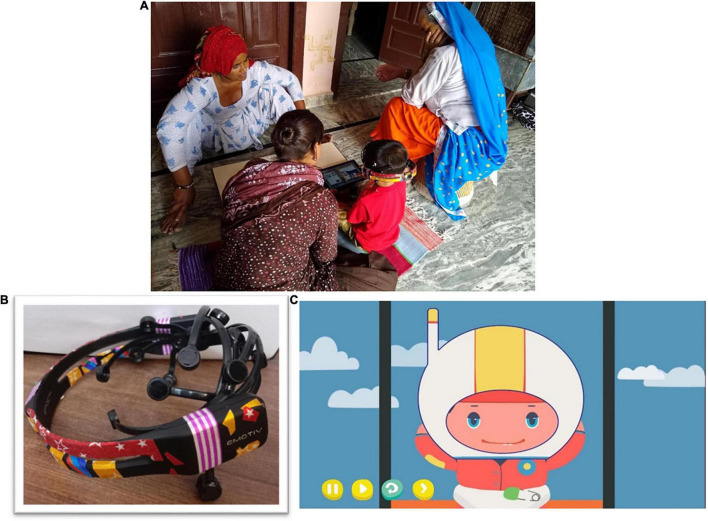
**(A)** Example picture of testing in rural households. **(B)** EMOTIV EPOC decorated with stickers to make it attractive for children. **(C)** Screenshot of the animated story showing the protagonist wearing a helmet, which was used to encourage children to wear the EEG headset.

In this study, EEG assessments were conducted by non-specialists (henceforth referred to as “assessors”) in participants’ households at a convenient date and time (Figure 1A). Assessors were prioritized for recruitment if they had prior training and experience working with young children and were from the same community in which the study was being conducted. All eight assessors had completed the equivalent of a postgraduate degree and none had worked with EEG technology before.

### Equipment

Electroencephalography was conducted using the wireless EPOC device by EMOTIV connected to Microsoft Surface Pro 4 tablets. It consists of 14 gold plated saline electrodes (sensors contained felt inserts) positioned by the 10–20 International system at AF3, AF4, F3, F4, F7, F8, FC5, FC6, T7, T8, P7, P8, O1, and O2. The EEG headsets use CMS/DRL reference system (M1, a ground reference point for measuring the voltage of the other sensors and M2, a feed-forward reference point for reducing electrical interference from external sources). The device has a maximum sampling rate of 128 Hz along with digital notch filters for line noise at 50 and 60Hz. The spectral range reliably available for analysis is 0.2–45 Hz. Impedance at each electrode is measured, color-coded and reported in real-time as “channel contact quality” ranging from 0 to 4. The EPOC device has a 12-h battery life, while the Microsoft Surface Pro 4 tablet battery life lasts approximately 4–5 h. Since re-using felt sensors across participants was not recommended for hygiene purposes, and EMOTIV’s proprietary felt inserts were prohibitively expensive for the scale of data collection planned in this study, they were replaced with 1-cm long dental cotton roll pieces. Further, in order to increase the acceptability of the EEG headset to 3-year-old children, the device was decorated with stickers to make it colorful and attractive (Figure 1B).

#### Criteria Guiding Selection of the EPOC Device

##### Prior Evidence

The EPOC device has been available for over a decade, and a recent review has examined its use in research studies (Williams et al., 2020). This review found that while most studies using the EPOC headset have been conducted with adults, some groups have begun exploring its potential for use with children over the age of 5 years, including those with special needs such as Autism (Askari et al., 2018) and Attention Deficit Hyperactivity Disorder (ADHD) (Martínez et al., 2016; Mercado-Aguirre et al., 2019). The utility of this device to collect EEG data and extract a variety of metrics ranging from power across spectral frequency bands such as alpha, beta and gamma in resting-state eyes open condition (Askari et al., 2018), as well as while children are engaged in a game targeting cognitive, attention and reasoning skills (Martínez et al., 2016); and event-related potentials (ERPs) elicited in response to auditory stimuli (Badcock et al., 2015; Mercado-Aguirre et al., 2019) has already been demonstrated.

##### Hardware Design

We expected that the design of the headset in a fixed configuration with all electrodes embedded within arms would contribute to the ease with which it could be accurately placed onto the head by non-specialists. Being saline sensors, it is less messy and does not leave a residue in the hair. The long battery-life of the headset would also make it possible to record data from multiple households in a day, without the need for access to electricity.

##### User-Friendly Software

The EMOTIV Pro data collection software can be used offline, and we expected that its user-friendly interface would aid non-specialists to collect EEG data. For example, the use of colors to indicate channel quality greatly facilitated training without the need to train on technical details of EEG signals such as impedance.

### Study Procedures

This study was conducted in two phases: (1) Formative phase in which contextually relevant standard operating protocols to conduct EEG research on children and adults in rural household settings was established and (2) Implementation phase in which the protocol established through formative work was assessed through implementation on a large sample of children and adults.

#### Formative Phase

The formative phase was conducted with 9 families between May and July 2018, who were recruited from four villages adjoining the SPRING study area. The children in these households (3 girls and 6 boys) were aged between 32 and 37 months. Every visit in this phase included a field supervisor (KKS) accompanying an assessor to make detailed observations to inform the following components of the standard operating protocol: consenting, engaging children in the EEG assessment, and data collection (observations and learnings are summarized in Table 1).

**TABLE 1:** Standard operating procedures informed by the formative phase of this study.

Study component	Challenges	Solutions
Consenting	Hesitancy due to unfamiliarity with EEG	Showing the EEG equipment to families and allowing them to hold and examine the device
		Showing small video clips of sample EEG assessments to family members
	Hesitancy in the mother to provide consent before consulting with other family members, especially the father	Ensuring that all available family members were involved in the consent process
		Leaving a collage of images illustrating the EEG assessment (see Supplementary Figure 1) in the household so absent family members could be informed about the assessment
Child engagement strategies	Children were intimidated by the headset	The EMOTIV EPOC device was decorated with stickers to make it colorful and attractive (see Figure 1B)
	Children hesitated to wear the headset	The animated video on the DEEP cognitive assessment tool (Bhavnani et al., 2019) contained a scene in which the protagonist (the child) wears a helmet-like headset before starting an exciting journey (see video snapshot in Figure 1B). Assessors played the video to engage the child, and paused it when the protagonist wore the helmet to encourage the participating child to wear a headset like the character in the video. They were told they could continue on their journey through the cognitive assessment game after wearing the headset
		The assessors wore the headset themselves, or requested the mother to do so briefly
Data collection protocols	Children were reluctant to close their eyes	Children were allowed to cover their eyes using their hands, or the caregiver or assessor could do so for them
	Due to the uncontrolled nature of the setting, disturbances were anticipated during data collection	Data collection was conducted in a separate room with only the primary caregiver(s) and the child in the household to minimize disturbances
		Assessors were trained to record the time stamps of any disturbance that led to visible changes in the EEG signal recording (example trace in Figure 2)

### Consenting

Field personnel observed a hesitancy amongst some families to participate in the EEG component of the study. The following strategies were thus incorporated into the consenting process to facilitate families’ understanding of the EEG assessment and associated hardware (see Table 1): (a) ensuring that all available family members, including family elders like grandparents, were listening to the description of the EEG component of the study, (b) taking the EEG headset along at the time of consenting to show it to families and allow them to hold and examine the device, (c) showing small video clips of EEG assessments to family members so they could visualize the process, and (d) leaving a leaflet containing a collage of images illustrating the EEG assessment (see Supplementary Figure 1) in the household so absent family members, such as fathers who were usually away at work, could be informed of what the assessment would entail.

### Child Engagement Strategies

A story, in the form of an animated video which is part of a cognitive assessment tool named Developmental Assessment on an E-Platform (DEEP) (Bhavnani et al., 2019), was used to encourage children to wear the EEG headset. In this video, the protagonist (a child) has to embark on a journey to help the moon reunite with its friends, the planets, and wears a helmet-like headset before starting the journey (see video snapshot in Figure 1C). Assessors were trained to pause the video at this point, and encourage the participating child to also wear a headset like the character in the video in order to continue on their journey through the cognitive assessment game. Modes of encouragement also included the assessors wearing the headset themselves, or requesting the mother to do so briefly.

### Data Collection Protocol

Unlike ERP studies, advantages of collecting data in resting-state include (a) capturing the default state of the brain and not stimulus triggered events, (b) the short duration of the recording, and (c) a lack of the requirement to synchronize across multiple devices typically used for stimulus presentation and data collection. Importantly, metrics derived from resting-state EEG signals have widely been demonstrated to index healthy brain development in cognitive, language, and socio-emotional domains in children and brain function in adults (Khanna et al., 2015; Anderson and Perone, 2018; Parameshwaran et al., 2019; Bhavnani et al., 2021). Thus a decision was taken to collect resting-state EEG data from adults and children with their eyes-closed and eyes-open. Additionally, EEG data was also collected while children engaged in the DEEP cognitive assessment tool. However, other than its inclusion in the tally of cumulative number of EEG recordings across the data collection period (see results), this dataset is not discussed further here. Separate recordings were made for each of these conditions.

**FIGURE 2:**
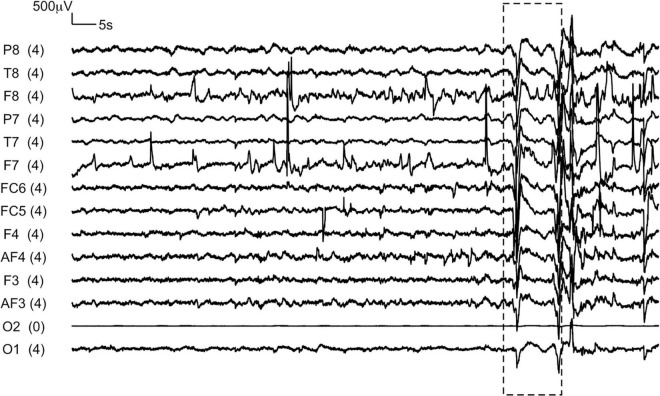
A representative EEG trace recorded from a child during eyes-closed resting state condition. The box represents the section of the recording during which the assessor reported a disruption due to a loud sound in the testing environment.

Since data was being collected in uncontrolled low-income household settings with chances of disturbance and interruption by other family members, the importance of testing participants in a relatively quiet part of the house and if available, in a separate room altogether, in order to avoid them being distracted during the assessment, was emphasized. In addition, the time stamps of any disturbance that visibly made the EEG recording fluctuate dramatically (example trace in Figure 2) was recorded by the assessor. It was observed that children were often reluctant to close their eyes, or they opened them after a few seconds. The protocol thus included the option for children to cover their eyes using their hands, or for the caregiver or assessor to do so for them.

#### Implementation Phase

The protocol developed in the formative phase (see column 3 in Table 1) was implemented in this phase. 1443 families from the SPRING study were approached to participate in this phase of the study conducted between August 2018 and March 2019. Of them, 178 were lost to follow up due to the following reasons: 122 had moved away from the study area, 40 were temporarily unavailable during the assessment period, 12 families refused consent, 3 children had died, and 1 child was unable to participate due to a physical handicap. The remaining 1,265 families were approached for EEG assessments on children and their parents. Only 9 families refused the EEG component due to concerns about potential adverse impact of the headset on the brain.

### Electroencephalography Training and Supervision

The training of non-specialist assessors to collect EEG data in children’s households was conducted over a day, split into two sessions and led by SB. The training was based on the EMOTIV EPOC and EMOTIV Pro software user manuals. Assessors were given a basic explanation of the brain’s ability to communicate through electrical signals and that brain electrical signals can be recorded using EEG electrodes. Since assessors were unfamiliar with EEG technology, this was compared conceptually to electrocardiograms (ECG) of which most were aware. Thereafter, they were given an introduction to the nomenclature of the components of the EPOC headset, its electrode locations and the importance of accurate placement of the headset on a head. This was done with the help of pictures from the manual and hands-on demonstrations on staff. Laminated pictures of the EEG electrode locations were given to assessors to carry with them and use as a reference when collecting data.

The assessors were then taught to operate the EMOTIV Pro software and connect the headset wirelessly to the Microsoft Surface Pro 4 tablet. In particular, their attention was drawn to the color-coding of each electrode on the software to demonstrate channel quality (CQ). Black color (CQ = 0) indicates no contact between the head and electrode. Colors red (CQ = 1), orange (CQ = 2), yellow (CQ = 3), and green (CQ = 4) indicated progressively better signal quality with “green” indicating impedance values below 20 kΩ (Williams et al., 2020). Assessors were trained to optimize channel quality by ensuring that the felt sensors/cotton rolls were appropriately soaked with saline solution and there was contact between the electrode and the surface of the head by gently moving hair away from the electrode location. They were trained to start the independent recording for each resting-state condition only when all the electrodes displayed green or yellow colors. Participants were requested to first keep their eyes closed for 3 min and then either keep them open and stare at a single stable point on the wall in front of them (adults) or, as is common practice in EEG studies with preschool children, make them watch a passive screensaver video of 3 min (Jensen et al., 2021). It was ensured that the assessors started each independent EEG recording a couple of seconds *after* the participant had settled down for each of the resting-state conditions (closed their eyes or the video had started).

This classroom training was followed by practice sessions in which the assessors gained experience in handling the headset by using it on each other and staff’s children. Once data collection began, weekly group meetings between the field supervisor and all assessors were used to provide peer support and regular feedback, and quarterly refresher trainings were conducted by the senior research team member (SB). The field supervisor (KKS) also provided supervision during the scale-up phase of the study.

### Electroencephalography Data Processing

Electroencephalography data was processed using analysis scripts created in-house by our team on R (RStudio Team, 2020) (R-script and metrics can be found on this link). Since numerous studies demonstrate that filtering can affect and distort the shape and temporal structure of EEG signals (Vanrullen, 2011; Acunzo et al., 2012), no filtering was done on the EEG signals obtained in this study except centering using mean correction. Characteristics of the acquired EEG signal to be analyzed were computed by power spectral density (PSD) estimation using Welch method (Parameshwaran and Thiagarajan, 2019). The PSD of the EEG signal is typically a decreasing decay function with lower power at higher frequencies. One characteristic of the PSD that is discussed in this study is the regular oscillatory activity in the Alpha band that appears as a peak above the background envelope of the PSD. The frequency corresponding to peak of this Alpha band activity (Pa) is typically, though not always, observed at around ∼7–11 Hz in adults and 6–9 Hz in children, most reliably under eyes-closed conditions and at electrodes located at O1 and O2 positions (Marshall et al., 2002; Parameshwaran and Thiagarajan, 2019; Webster and Ro, 2020). The percentage of participants which had an alpha peak at each electrode position was calculated. The other simple characteristic of the EEG time series discussed here includes the standard deviation of the amplitude distribution [A_SD = std (EEG time series)].

### Data Collection on Participant Characteristics

#### Socioeconomic Status

Information on parental education and socioeconomic status (SES) was collected from families when they first enrolled in the SPRING study in 2014/15 (Bhopal et al., 2019a). Principal components analysis was used to calculate an SES index using data on household demographics and animal and other asset ownership. This index was used to categorize the population into SES quintiles.

#### Preschool Attendance

During the visit conducted at 3 years age as part of this study, parents were asked if their child attended any public or private preschool.

#### Anthropometry

World Health Organization (WHO) protocols (The WHO Child Growth Standards, n.d.) were used to measure the child’s height using the Seca 213 Portable Stadiometer and weight using the SECA-384 electronic scale. Height and weight were used to generate height-for-age (HAZ) and weight-for-age (WAZ) z-scores using WHO growth standards. Head circumference was measured using SECA-201 measuring tape.

Continuous and categorical data were compared using Student’s t-tests and chi-square tests respectively on STATA version 12^4^.

We have uploaded the EDF files, EEG metrics and associated metrics on Brainbase. We are unable to publicly share the.edf files since they are linked to identifiable information. However, this data can be shared with interested parties on a case-by-case basis upon receipt of a reasonable request and exchange of data sharing agreements.

## Results

### Description of Study Participants

Of the 1256 families who consented, resting-state EEG data was collected from 977 children (N_eyes–open_ = 967, N_eyes–closed_ = 914, N_both_ = 904). The children had a mean age of just over 3 years (38.8 months, standard deviation (SD) = 0.9 months) and 46.1% were girls (Table 2). The mean head circumference of participating children was 47.7cm (SD = 1.4 cm), 32% were stunted and 25.7% were underweight defined as height-for-age (HAZ) and weight-for-ae (WAZ) being over two standard deviations less than the mean as per WHO growth standards. Over half the children (53.6%) did not attend any formal preschool. Resting-state EEG data was also collected from 1199 adults (1068 mothers and 131 fathers of participating children, mean age = 27 years, SD = 4 years) (N_eyes–open_ = 1194, N_eyes–closed_ = 1190, N_both_ = 1054) (Table 3).

**TABLE 2:** Participant demographics.

	Children with any EEG data (*N* = 977)	Children without EEG data (*N* = 288)	t/chi^2^ (*p* value)
Age of child in months, mean (SD)	38.8 (0.9)	39.0 (1.0)	2.7 (0.006)
Female, *n* (%)	450 (46.1%)	125 (43.4%)	0.6 (0.43)
HAZ, mean (SD) Stunted, %	−1.5 (1.0) 32	−1.6 (1.0) 34	−1.4 (0.15) 0.4 (0.53)
WAZ, mean (SD) Underweight, %	−1.4 (1.0) 25.7	−1.5 (0.9) 29.2	−1.5 (0.13) 1.4 (0.24)
Head circumference in cm, mean (SD)	47.7 (1.4)	47.5 (1.4)	−1.4 (0.15)
Preschool enrollment, % Not attending Private Anganwadi centers	53.6 27 19.3	64.2 16.7 19.1	14.1 (0.001)
Mother’s education level, %* Below primary (including never been to school) Primary/middle school completed Secondary/higher secondary school completed College and above	11.68 26.33 38.63 23.36	12.85 25.35 38.89 22.92	2.1 (0.91)
Father’s education level, %* Below primary (including never been to school) Primary/middle school completed Secondary/higher secondary school completed College and above	5.53 19.57 45.08 29.81	4.51 17.36 47.22 30.9	5.9 (0.43)
SES quintile (during enrollment), %* Q1 (poorest) Q2 Q3 Q4 Q5 (wealthiest)	20.6 24.0 19.2 20.4 15.9	20.5 18.4 22.9 16.0 22.2	12.0 (0.02)

*1 missing data.

**TABLE 3:** Study component characteristics.

	Child		Adult	
	Eyes open	Eyes closed	Eyes open	Eyes closed
Sample size, *N*	914	967	1190	1194
Duration of recording (sec), mean (sd)	163.2 (17)	81 (63)	183.5 (15)	182.3 (9.5)
Channel quality, mean (SD)	3.8 (0.24)	3.79 (0.26)	3.78 (0.26)	3.97 (0.11)
Presence of peak alpha (%)	48.2	59.5	57.1	71.3
Frequency of peak alpha (Hz), mean (SD)	8.19 (1.95)	8.43 (1.73)	10.73 (0.26)	10.71 (3.96)

### Acceptability of Electroencephalography Assessments to 3-Year-Old Children

Of the 1,256 families who consented for EEG assessments, 977 children (77.8%) provided resting-state data for this study. 259 children (20.6%) refused to wear the EEG cap or got upset and removed it after wearing it. Hardware or software issues were experienced during 30 and 25 recordings in eyes-open and eyes-closed conditions respectively, resulting in loss of EEG data. 58 children (4.6%) wore the cap but refused the eyes-closed recording session. The demographic data of children who did not contribute any resting-state EEG data to this study for the reasons outlined above were compared to those who did (Table 2). A larger proportion of children that provided EEG data were attending preschool (27% compared to 16.7% of those who didn’t provide EEG data, *p* = 0.001), and there were fewer children from the wealthiest SES quintile (15.9% in Q5 compared to 22.2%, *p* = 0.02). Children in the two groups did not differ significantly in any other key indicators such as height- and weight-for-age and highest level of parental education.

### Feasibility of Electroencephalography Data Collection in Rural Households

A significant challenge in household-based data collection from adults was the unavailability of fathers due to work commitments. Assessors observed participants move their heads or touch the EEG headset with their hands during the assessment in 78 of the 1881 resting-state recordings (4.1%) from children, and 40 of the 2384 recordings (1.7%) from adults. Assessors also reported that 59 of the 915 children (6.4%), and 4 of the 1190 (0.3%) adults, who contributed to the eyes-closed condition had opened their eyes for a few seconds within the recording duration. Assessors reported visible changes in the EEG signal as a result of disruptive sounds in the testing environment, for example family members speaking or sound from gadgets like mobile phones, TVs or radios, only for 23/5223 total recordings (0.4%). Figure 2 shows a representative EEG trace in which the presence of such disruptive sounds in the testing environment toward the end of the recording, as reported by the assessor, is visible (box).

### Utility of the Electroencephalography Recording

**FIGURE 3:**
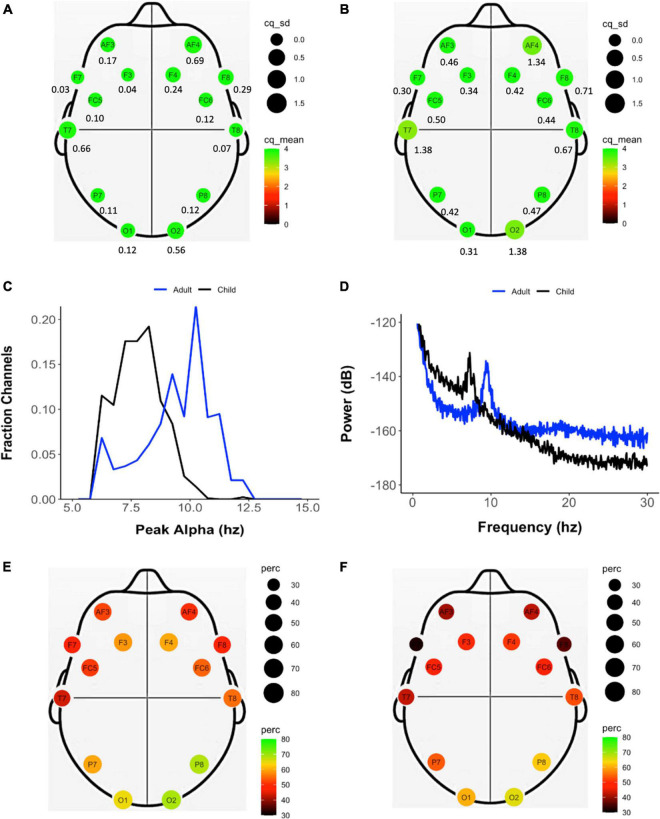
Properties of the EEG data recorded during eyes-closed resting state across the study sample of adults and children. Mean (color and size coded) and standard deviation (reported below each electrode location) of the channel quality of each electrode in adults **(A)** and **(B)** children. **(C)** Frequency of peak alpha (Pa) calculated individually for each channel in adults (blue) and children (black). **(D)** Power spectrum derived from representative EEG recordings from adults (blue) and children (black). Frequency (color and size coded) of presence of an alpha peak at each electrode location in adults **(E)** and children **(F)**.

The properties of the EEG recordings obtained in this study are summarized in Table 3. As per the protocol, assessors attempted to record EEG in eyes-open and eyes-closed conditions for 3 min each. The mean duration of the eyes-closed EEG recordings were 1.35 min (SD = 1.05 min) in children and 3.06 min (SD = 15 s) in adults. In eyes-open condition, the mean recording duration was 2.72 min (SD = 17 s) in children and 3.04 min (SD = 9.5 s) in adults. The mean and SD of channel quality for each electrode in eyes-closed condition in adults and children can be found in Figures 3A,B and Supplementary Table 1. Apart from electrodes at positions AF4, T7, and O2 (Figure 2 shows an example trace in which the O2 was not working at all), where channel quality varied across participants, very high channel quality with low standard deviation was obtained throughout the study (ranging from 3.29 to 3.96 in children and 3.86 to 3.99 in adults on a scale of 0–4). Also, adults showed consistently higher channel quality compared to children, possibly due to the headset size being more suitable for them. In both children and adults, the percentage of recordings which showed an alpha peak (Pa) was higher in eyes-closed than eyes-open condition (Table 3). The mean frequency of the alpha peak was found to be at 8.43 Hz (SD = 1.73) in children and 10.71 Hz (SD = 3.96) in adults (Figure 3C). The power spectrum of an illustrative eyes-closed recording from children and adults demonstrating the frequency of Pa can be found in Figure 3D. Figures 3E,F show that the electrode position at which the alpha peak occurred most frequently was O1 and O2 in both children and adults.

**FIGURE 4:**
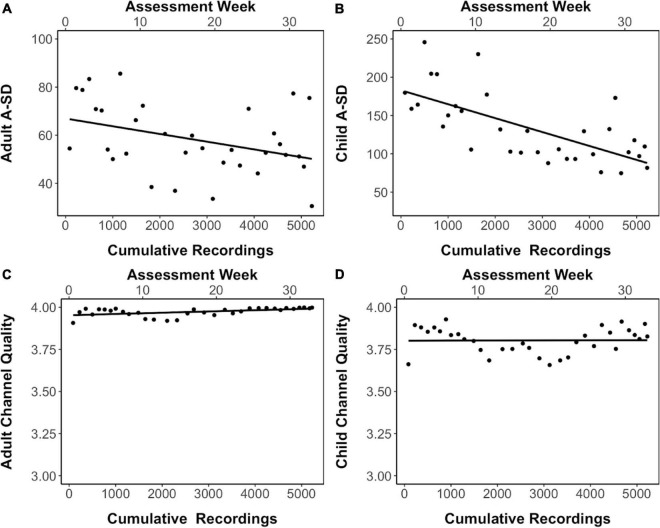
Signal quality in eyes-closed resting-state EEG recordings. **(A,B)** The standard deviation of the amplitude of the EEG signal (A_SD) in adults and children respectively across data collection period and increased device usage. **(C,D)** The channel quality across data collection period and increased device usage in adults and children respectively. Linear trendline is shown.

Electroencephalography data collection in this study occurred over a 33-week duration with four devices being used simultaneously by the four teams of assessors to collect data. The number of EEG recordings, including eyes-open and eyes-closed in children and adults and gameplay in children, which were made across this period totaled to 5223 recordings. As sensor contact quality decreases, the signal decreases in resolution which results in reduced variance of the amplitude and fluctuations. The standard deviation of the amplitude (A_SD) of the EEG signal derived from adults and children can thus be considered to be a proxy measure for sensor sensitivity (Liu et al., 2019). The average A_SD obtained across all sensors in the four devices in an assessment week decreased as the study progressed and the number of cumulative recordings done on these devices increased (Figures 4A,B), indicating that sensor quality may have degraded with increasing use. Interestingly, channel quality, which is an indirect readout of impedance from the data collection software, did not show this decline across the data collection period and increased device usage (Figures 4C,D).

## Discussion

In this study we present the acceptability, feasibility and potential utility of conducting resting-state EEG assessments on preschool children and adults in household settings in rural north India. To our knowledge, this is the first published study to use a relatively low-cost EEG portable device administered by non-specialists to collect EEG data from children in over 900 rural households.

### Acceptability of Electroencephalography to Families and Preschool Children

While EEG has been used in laboratory and clinical settings for decades, its application outside of these settings remains a novelty, especially in rural households in LAMIC. Despite this, our study shows high levels of acceptability as demonstrated by the low rate (0.7%) of consent refusal by families for this study component. This might be reflective of the embedding of the study team in the community for over six years at the time of data collection since the beginning of the SPRING study. It might also reflect the advantages of collecting data in households, as opposed to clinics or community centers where participants are required to travel to a different place for assessments. In a different study where we conducted a qualitative exploration of acceptability of EEG data collection in community centers in low-income settings in Delhi, mothers of participating children highlighted that logistical challenges associated with transportation of participants from households to community centers served as a barrier to participation (Lockwood Estrin et al., 2021^5^). We thus believe that collecting EEG data in households reduced the burden on families and facilitated their participation in this study.

In most EEG studies, particularly those with preschool children, a common reason for small samples is the attrition of participants due to children’s refusal to wear the EEG headset or adhere to the EEG data collection protocol, which occurs 30–45% of the time (Bell and Cuevas, 2012). The attrition rate of 20% found in this study is substantially better than that reported in laboratory studies conducted with 3-year-old children (Wolfe and Bell, 2007; Morasch and Bell, 2011; Cuevas et al., 2012). We hypothesize that conducting these assessments in the comfort of the child’s own home, and embedding the wearing of the EEG-like headset by the protagonist within the animated story which children were made to watch, facilitated this high rate of acceptability to children. Further, children were motivated to participate in the data collection protocol of keeping their eyes closed by the fact that it was to be followed by them playing a computerized game on a tablet computer (DEEP), which was highly novel and engaging to them (Bhavnani et al., 2019). Established researchers in the field of developmental EEG research also acknowledge how critical motivations such as toys and computer games are useful in engaging pre-schoolers in assessment protocols (Bell and Cuevas, 2012). However, we observed that while we were able to obtain approximately 3-min long EEG recordings from both children and adults in eyes-open conditions, children often did not keep their eyes closed for the entire 3-min duration while adults did. Despite this, the relatively short duration of one and a half minutes of eyes-closed data collected seems to be enough to extract metrics which demonstrate the potential utility of these recordings (see below).

### Feasibility of Electroencephalography Data Collection at Scale in Rural Households by Non-specialists

In this study, we demonstrate that it is feasible for non-specialists to collect EEG data with a one-day classroom training. We speculate that the equipment, software and protocol used in this study allowed us to make a critical advance to the methods of other studies conducted in other low resource settings in which EEG data collection was done by relatively technically advanced research personnel such as Ph.D. students, post-doctoral fellows or laboratory technicians (Storrs, 2017; Katus et al., 2019). In this study, we demonstrate that it is feasible for EEG to be administered by non-specialists naive to EEG technology, specifically, local women recruited from the community in which the study was conducted.

One of the main challenges associated with data collection in household settings is testing in an uncontrolled environment with potential for various types of loud sounds, movement, and electrical disturbances. Interestingly, disturbances that led to visible changes in the EEG signal that prompted assessors to make a note were extremely rare. This might be because the duration of the resting-state recordings was relatively short. Additionally, assessor training on the protocol of household visits included the importance of testing participants in a separate room within the household to minimize chances of any distractions during the assessment. We speculate that the few reported disturbances, thus, might represent circumstances within specific household settings such as the inability to conduct testing in a separate room, or the house being located in a noisy environment.

While recognizing the advantages of the low-cost, portable EMOTIV EPOC headset and data collection software, described in detail above, this study has also highlighted some of its important limitations, particularly with respect to large-scale data collection. The deterioration of signal observed due to excessive usage presents one such limitation. This might be due to the metallic electrode gradually getting oxidized over time due to excessive exposure to saline. Thus, although replacement for sensors are available, since the socket holding the sensor or the threading got damaged, over time, this led to an increase in impedance and attenuation of signal range (Kappenman and Luck, 2010). Importantly though, this increase in impedance did not reflect a change in channel quality as reported by the data collection software, which might be due to a relatively wide range of impedance values being categorized into each of the four possible channel quality output values. While broad categorizations aid in simplifying the user interface and its interpretability, it can compromise the precision with which small changes in device performance can be detected, ultimately affecting the quality of data available across large samples. In addition, while the fixed configuration of the headset had advantages with respect to training non-specialists, its disadvantages included the inability to replace individual faulty or oxidized electrodes. Given our experience in this study, we speculate that each device can be used to collect between 350 and 500 good quality EEG recordings, with constant careful attention being paid to the condition of the headset and sensor as usage progresses. We further recommend that researchers carefully consider the amount of usage of the EEG device, and the possible need for its replacement within the duration of a study, while estimating the cost-benefit ratio of using such low-cost EEG equipment. These findings also highlight the need to study other low-cost EEG data collection systems which might be more durable, like gel-based devices, to test their use at scale.

An important characteristic of the spectral properties of the EEG trace is the presence of regular oscillatory activity in eyes-closed conditions. The power spectrum of EEG signals derived from the EMOTIV EPOC in this study shows the presence of an alpha peak more often in eyes-closed than eyes-open resting state condition, and most frequently in electrodes located at O1 and O2 positions, which are both well-established properties of this EEG metric (Gale et al., 1971). Additionally, the mean frequency of the peak shifts from 8.43 Hz in children and 10.71 Hz in adults, similar to findings from laboratory studies in these populations (Marshall et al., 2002; Perone et al., 2018), providing evidence of the usability of this dataset.

### Strengths and Limitations

The strengths of this study include (a) data collection on a large sample of preschool children and their adult parents, (b) the use of a portable data collection system comprising a relatively low-cost EEG device connected to a tablet computer, (c) the use of non-specialist workers to collect EEG data, and (d) the collection of EEG data in uncontrolled settings of rural households. One important limitation of our study is the use of the EMOTIV EPOC, which has been designed for use on adult head sizes, on children who have a smaller head circumference. We speculate that the use of child-size devices would significantly improve the quality of the EEG signal. We also did not use any lab-grade EEG device implemented by specialist providers concurrently with the EMOTIV EPOC, to compare the latter’s performance in our setting.

## Conclusion

There is an urgent need to test and validate technological advances in neurophysiological methods, such as low-cost scalable EEG devices, for their potential to collect large scale data from young children and adults in populations which are historically underrepresented in such research, while arguably being the most likely to benefit from it. This study demonstrates the acceptability and feasibility of conducting such EEG studies at scale in a rural low-resource community and provides some preliminary insights into the basic characteristics of the EEG data derived. A key next step of this work is to explore the potential of metrics derived from this resting-state EEG dataset to index cognitive development and function in this population of preschool children and adults. Our results offer the impetus needed to further refine the methods and devices and validate such scalable methods to overcome existing research inequity. These are the essential first steps toward gaining a comprehensive understanding of the diversities in brain development and function across populations and settings, based on the evidence generated from samples that have traditionally been underrepresented or missing in EEG research due to logistical challenges.

## Data Availability Statement

The raw data supporting the conclusions of this article will be made available by the authors, without undue reservation.

## Ethics Statement

The studies involving human participants were reviewed and approved by Institutional Review Board, Sangath and Institutional Ethics Committee, Public Health Foundation of India. Written informed consent to participate in this study was provided by the participants’ legal guardian/next of kin. Written informed consent was obtained from the individual(s), and minor(s)’ legal guardian/next of kin, for the publication of any potentially identifiable images or data included in this article.

## Author Contributions

SB, DP, DM, GD, TT, and VP were responsible for study conception and design. SB, DP, KS, and DM were responsible for the acquisition and management of data. SB, DP, DM, TT, and VP analyzed and interpreted the data. SB, DP, and DM drafted the manuscript. All authors edited and approved the manuscript.

## Conflict of Interest

The authors declare that the research was conducted in the absence of any commercial or financial relationships that could be construed as a potential conflict of interest.

## Publisher’s Note

All claims expressed in this article are solely those of the authors and do not necessarily represent those of their affiliated organizations, or those of the publisher, the editors and the reviewers. Any product that may be evaluated in this article, or claim that may be made by its manufacturer, is not guaranteed or endorsed by the publisher.

## Footnotes

1
www.emotiv.com

2
www.cgxsystems.com/

3
openbci.com

4
https://www.stata.com

5 Estrin, G. L., Bhavnani, S., Goodwin, A., Arora, R., Divan, G., Haartsen, R., et al. (2021). From the lab to the field: acceptability of using electroencephalography with Indian preschool children.
