# Supplementary material for: The Acceptability, Feasibility, and Utility of Portable Electroencephalography to Study Resting-State Neurophysiology in Rural Communities
Source: Front Hum Neurosci. 2022 Mar 21;16:802764. doi: 10.3389/fnhum.2022.802764 (PMC8978891; doi:10.3389/fnhum.2022.802764)
Supplement: Supplementary file 1 [file Table_1.DOCX]

Supplementary Material

# Supplementary Figures and Tables


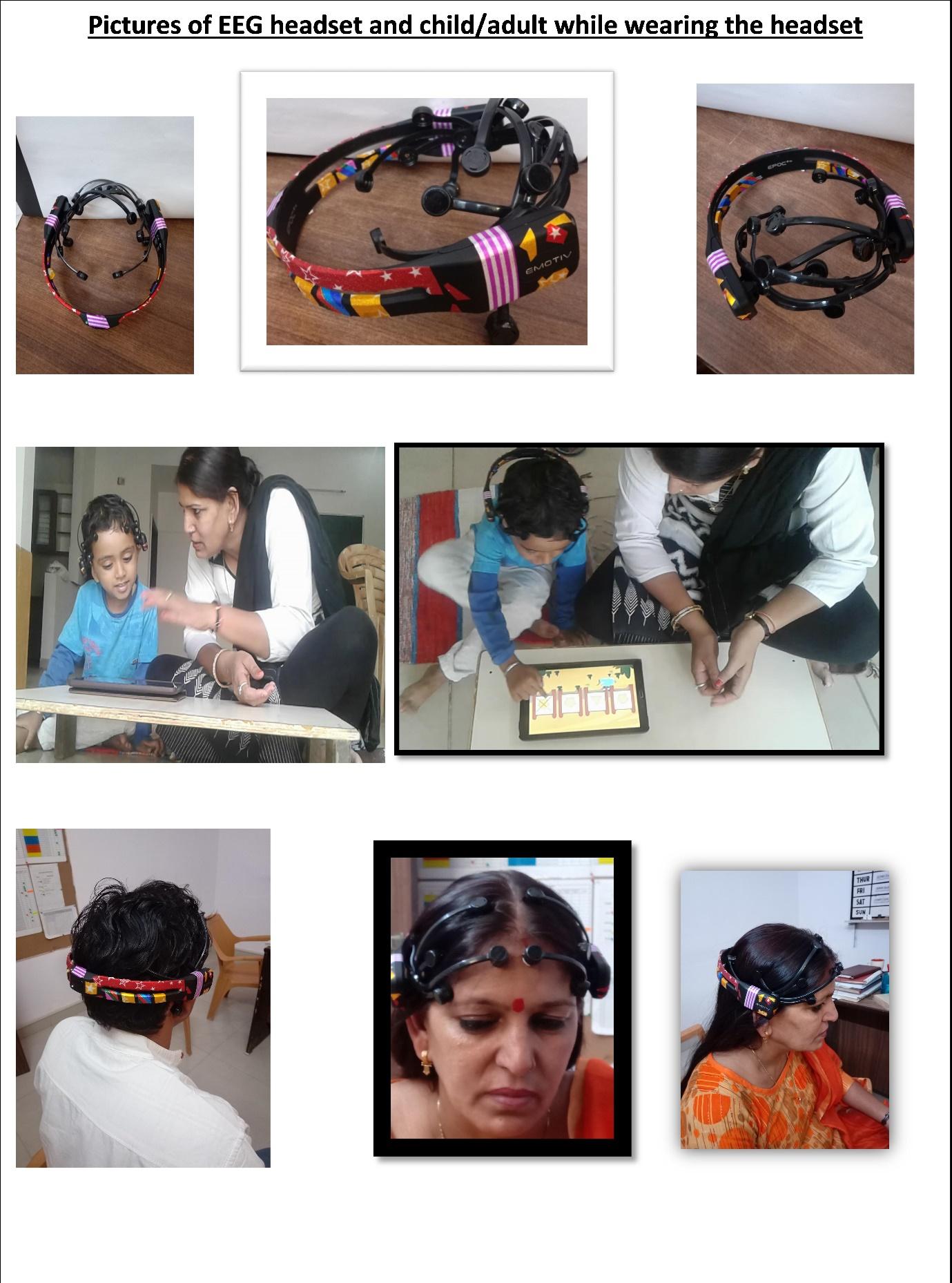


Supplementary Figure1: Leaflet used as an aid to explain EEG to families during consenting

Supplementary Table1: Mean and standard deviation of channel quality obtained during eyes-closed resting state recordings for each electrode in children and adults

| Channel | Channel Quality (CQ) in children, mean (standard deviation) | Channel Quality (CQ) in adults, mean (standard deviation) |
| --- | --- | --- |
| AF3 | 3.91 (0.46) | 3.99 (0.17) |
| AF4 | 3.43 (1.34) | 3.87 (0.69) |
| F3 | 3.94 (0.34) | 3.99 (0.04) |
| F4 | 3.93 (0.42) | 3.98 (0.24) |
| F7 | 3.96 (0.30) | 3.99 (0.03) |
| F8 | 3.85 (0.71) | 3.97 (0.29) |
| FC5 | 3.89 (0.50) | 3.99 (0.10) |
| FC6 | 3.92 (0.44) | 3.99 (0.12) |
| O1 | 3.95 (0.31) | 3.99 (0.12) |
| O2 | 3.37 (1.38) | 3.90 (0.56) |
| P7 | 3.92 (0.42) | 3.99 (0.11) |
| P8 | 3.91 (0.47) | 3.99 (0.12) |
| T7 | 3.29 (1.38) | 3.86 (0.66) |
| T8 | 3.86 (0.67) | 3.99 (0.07) |
